# RhoGAPp190: A potential player in tbph-mediated neurodegeneration in *Drosophila*

**DOI:** 10.1371/journal.pone.0195845

**Published:** 2018-04-13

**Authors:** Simona Langellotti, Giulia Romano, Fabian Feiguin, Francisco Ernesto Baralle, Maurizio Romano

**Affiliations:** 1 International Centre for Genetic Engineering and Biotechnology, Trieste, Italy; 2 Department of Life Sciences, University of Trieste, Trieste, Italy; Biomedical Sciences Research Center Alexander Fleming, GREECE

## Abstract

TDP-43 is an ubiquitous and highly conserved ribonucleoprotein involved in several cellular processes including pre-mRNA splicing, transcription, mRNA stability and transport. Notwithstanding the evidence of TDP-43 involvement in the pathogenesis of different neurodegenerative disorders (i.e. ALS and FTLD), the underlying mechanisms are still unclear. Given the high degree of functional similarity between the human and fly orthologs of TDP-43, *Drosophila melanogaster* is a simple and useful model to study the pathophysiological role of this protein *in vivo*. It has been demonstrated that the depletion of the TDP-43 fly ortholog (tbph) induces deficient locomotive behaviors and reduces life span and anatomical defects at the neuromuscular junction. In this study, using the known binding specificity of TDP-43/tbph for (UG) repeated sequences, we performed a bioinformatic screening for fly genes with at least 6 (TG) repeats in a row within the 3'-UTR regions in order to identify the genes that might be regulated by this factor. Among these genes, we were able to identify RhoGAPp190 as a potential target of the tbph-mediated neurodegeneration. RhoGAPp190 is a negative regulator of *Drosophila* RhoA, a GTPase protein implicated in the fine modulation of critical cellular processes including axon branch stability and motor axon defasciculation at muscle level and cognitive processes. We were able to demonstrate that the RhoGAPp190 expression is upregulated in a tbph-null fly model, providing evidence that this deregulation is associated to tbph silencing. Our results introduce RhoGAPp190 as a novel potential mediator in the complex scenario of events resulting from *in vivo* tbph loss-of-function.

## Introduction

TDP-43 is an ubiquitously expressed nuclear factor, implicated in several aspects of cellular biology including mRNA splicing, transport and stability [1]. In 2006, it was identified as the main protein component of intracellular inclusions observed in neurons and glial cells of patients affected by ALS and FTLD [2, 3]. In spite of a growing body of evidence supporting the involvement of TDP-43 in the pathogenesis of several neurodegenerative disorders [4, 5], the underlying molecular mechanisms have not been yet completely clarified. Ex-vivo and in-vitro studies strongly suggest that the cytoplasmic and nuclear inclusions might trigger gradual entrapment of endogenous TDP-43 in an insoluble and non-functional form, leading to the progressive impairment of its physiological activities [6, 7]. TDP-43 is evolutionarily conserved across many species and a considerable level of functional homology has been also demonstrated [8–10]. The characterization of the molecular mechanisms of TDP43-mediated neurodegeneration relies on identification of suitable animal models expressing funct ionally homologues of the human TDP-43. In this line of work, the *Drosophila melanogaster* ortholog of TDP-43 (namely, tbph) shares a number of structural and functional features with the human protein. Not only tbph is able to preferentially bind the same (UG)n rich sequences [8], it is also able to repress splicing of specific exons targeted by the human ortholog [8, 11]. Thus, the availability of TDP-43-null animal models is of great relevance to gain better insight into the molecular mechanisms potentially related with the neurodegeneration derived by TDP-43 loss-of-function [4, 12, 13]. In order to identify genes whose expression is controlled by tbph, we decided to perform a bioinformatic screening for fruit fly genes that could possibly be regulated by this nuclear factor and subsequently validate the "in silico" results by performing in vivo and in vitro experiments designed to reveal the genetic and physical interaction (at mRNA level) between tbph and the potential target genes. To this aim, we have focused our attention on the 3'UTR, as it is the site where many interactions influence mRNA stability and efficiency of translation.

## Results and discussion

### Identification of *Drosophila melanogaster* genes containing (TG) repeats within their 3'-UTR regions

The first step in our investigation on the role of TDP-43 in the pathogenesis of neurodegeneration was to identify genes whose expression could be influenced by this nuclear factor. Given the known binding specificity of TDP-43/tbph for (UG) repeated rich sequences [14], we sought to find fruit fly genes containing at least 6 (TG) repeats in a row within their 3’UTR region. This cut-off point was chosen because it has been demonstrated that TDP-43 binds stably RNAs containing >6 (UG) repetitions. Thus, using the BioMart system, we analyzed the collection of *Drosophila melanogaster* genes (BDGP6) in the ENSEMBL Genes 86 database by selecting (among attributes) the search for sequences of the 3'-UTR of the transcripts. The database interrogation returned 29173 entries. After the removal of 3401 items whose sequence was unavailable, the remaining records were sorted by the number of (TG) repetitions (ranging between 15 and 6). Table 1 shows the number of identified transcripts. Subsequently, we tested the expression of 15 genes selected amongst those containing the longer (TG) stretches (Table 2). S1 Table includes the complete list of all the genes identified.

**Table 1:** Transcripts and genes containing unique (TG)n within their 3’UTRs.

(TG)n	Transcripts	Genes
6	70	26
7	38	18
8	10	6
9	21	10
10	5	3
11	1	1
12	1	1
13	0	0
14	1	1
15	3	1

**Table 2:** Drosophila melanogaster genes containing (TG)_≥6_ within the 3'UTR tested by Real Time PCR.

FlyBase ID	Gene	Symbol	(TG)n	Additional (TG)n	FlyBase gene name
FBgn0261041	CG12295	stj	15	5	straightjacket
FBgn0000711	CG2096	flw	14		flapwing
FBgn0030434	CG4400		12		
FBgn0010329	CG1543	Tbh	11		Tyramine beta hydroxylase
FBgn0085426	CG34397	Rgk3	10		Rgk3
FBgn0263005	CG43313		10		
FBgn0000459	CG9908	disco	10		disconnected
FBgn0026375	CG32555	RhoGAPp190	9	7	RhoGAPp190
FBgn0027546	CG4766		9		
FBgn0010014	CG4209	CanB	9		Calcineurin B
FBgn0041342	CG4354	Cct1	9		CTP:PC cytidylyltransferase 1
FBgn0053517	CG33517	D2R	8	6	Dopamine 2-like receptor
FBgn0029713	CG11436		8		
FBgn0024184	CG6269	unc-4	8		unc-4
FBgn0039000	CG6954		8		

The FlyBase ID, Annotation symbol, Symbol, and Flybase gene name for each *Drosophila melanogaster* gene are indicated.

### RhoGAPp190 mRNA is upregulated in flies lacking tbph

**Fig 1:**
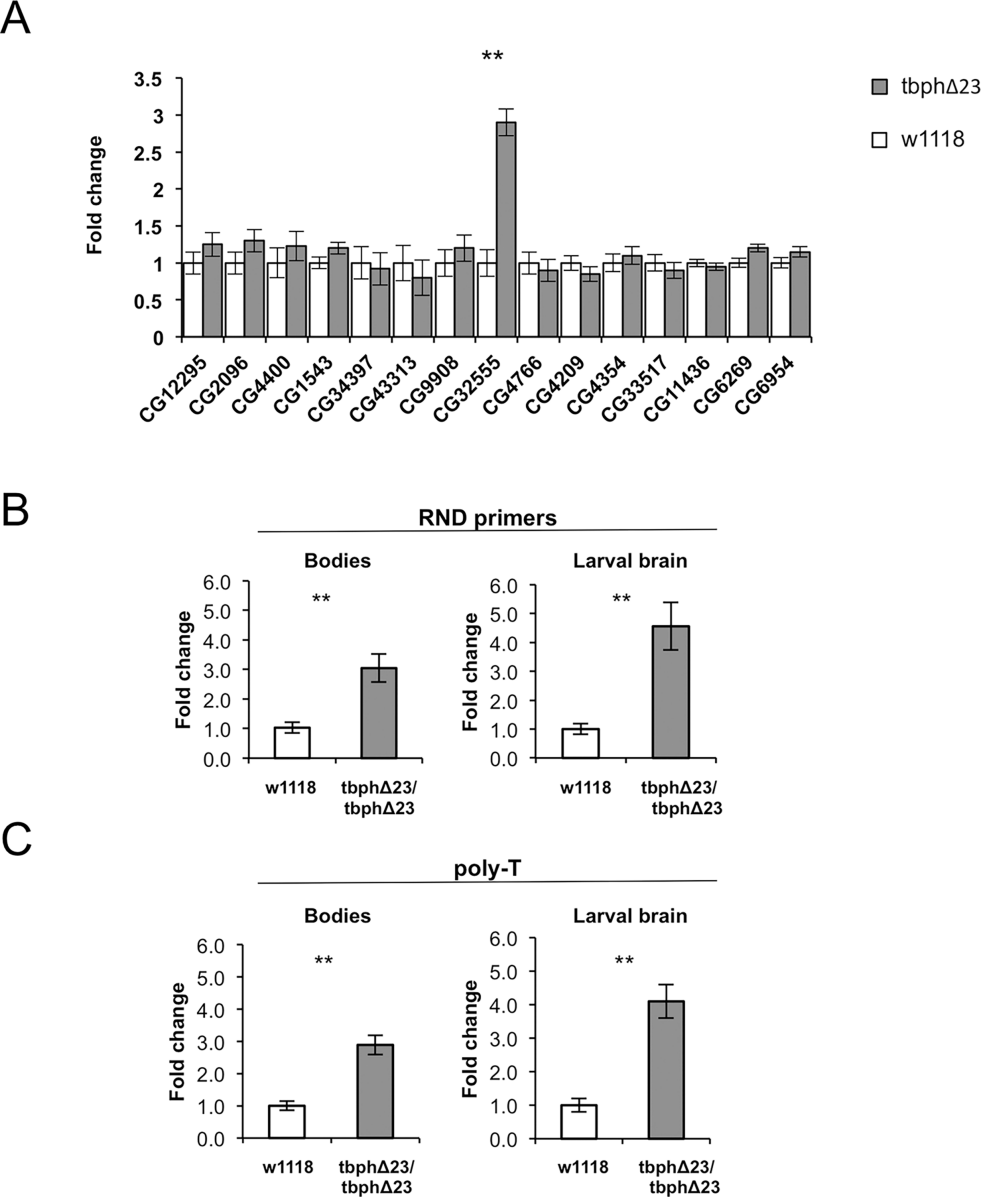
3'UTR-TG-gene expression comparison in tbph-null and control flies. A) Real Time PCR on RNA from fly heads samples: only the RhoGAPp190 mRNA (gene ID: CG32555) is significantly upregulated in the heads of tbph-null flies (tbphΔ23 genotype, gray bars) in comparison to controls (w1118 genotype, white bars). N = 3, **p<0.01 (t-test), error bars indicate SEM. The transcript’s expression difference was not statistically significant for the other tested genes. B) Real Time PCR carried out by using cDNA synthesized with Random primers (RND primers) or C) Real Time PCR carried out by using cDNA synthesized with poly-T primers (poly-T). Bodies samples: qPCR results show that RhoGAPp190 mRNA levels are upregulated also in the body of tbph-null flies. Larval brain: qPCR results show that RhoGAPp190 mRNA levels are upregulated also in the brain of tbph-null larvae (tbphΔ23), always in comparison to the w1118 line (w1118) used as a control. W1118 genotype, white bars; tbphΔ23 genotype, gray bars. Y-axis shows the relative fold change (tbphΔ23/w1118). N = 3, **p<0.01 (t-test), error bars indicate SEM.

In order to verify if tbph is actually able to influence the expression of these genes, we compared their expression levels in the w1118 line (used as a control) and a previously generated tbph-null allele fly model (tbphΔ23/tbphΔ23) [15]. Starting out from the observation that tbph is physiologically expressed at higher levels in *Drosophila* heads than in bodies [15], this screening was initially carried out by analyzing transcripts expression in fly heads (Fig 1A). Real Time PCR (qPCR) experiments revealed that RhoGAPp190 (gene ID CG32555) mRNA expression is significantly higher in the tbphΔ23 line, in comparison to w1118 flies (Fig 1A). Subsequently, to characterize the relationship between tbph and RhoGAPp190, we extended the tests to the fly bodies. The upregulation of this target gene was also observed in the bodies (Fig 1B and 1C, left panels). In addition, considering that the tbph-null flies present severe locomotor impairment already during larval stage [11, 16], we analysed whether the deregulation of RhoGAPp190 was detectable in tbph-null larvae or, on the contrary, solely during adulthood. We then analyzed RhoGAPp190 expression in brains dissected from third instar larvae and found a significant upregulation of this transcript at this developmental stage (Fig 1B and 1C, right panels). The presence of quantitation artifacts can be reasonably excluded by observing similar qPCR results using complementary DNAs (cDNAs) synthesized either with random primers (Fig 1B) or with oligo-dT (Fig 1C) primers. These results demonstrate that the RhoGAPp190 mRNA levels are upregulated in a *Drosophila* model lacking tbph expression, thereby suggesting that this nuclear factor could be essential to keep RhoGAPp190 expression finely controlled in physiological conditions. According to this hypothesis, the tbph protein might negatively regulate the RhoGAPp190 mRNA steady state levels.

### Genetic interaction between RhoGAPp190 and tbph

**Fig 2:**
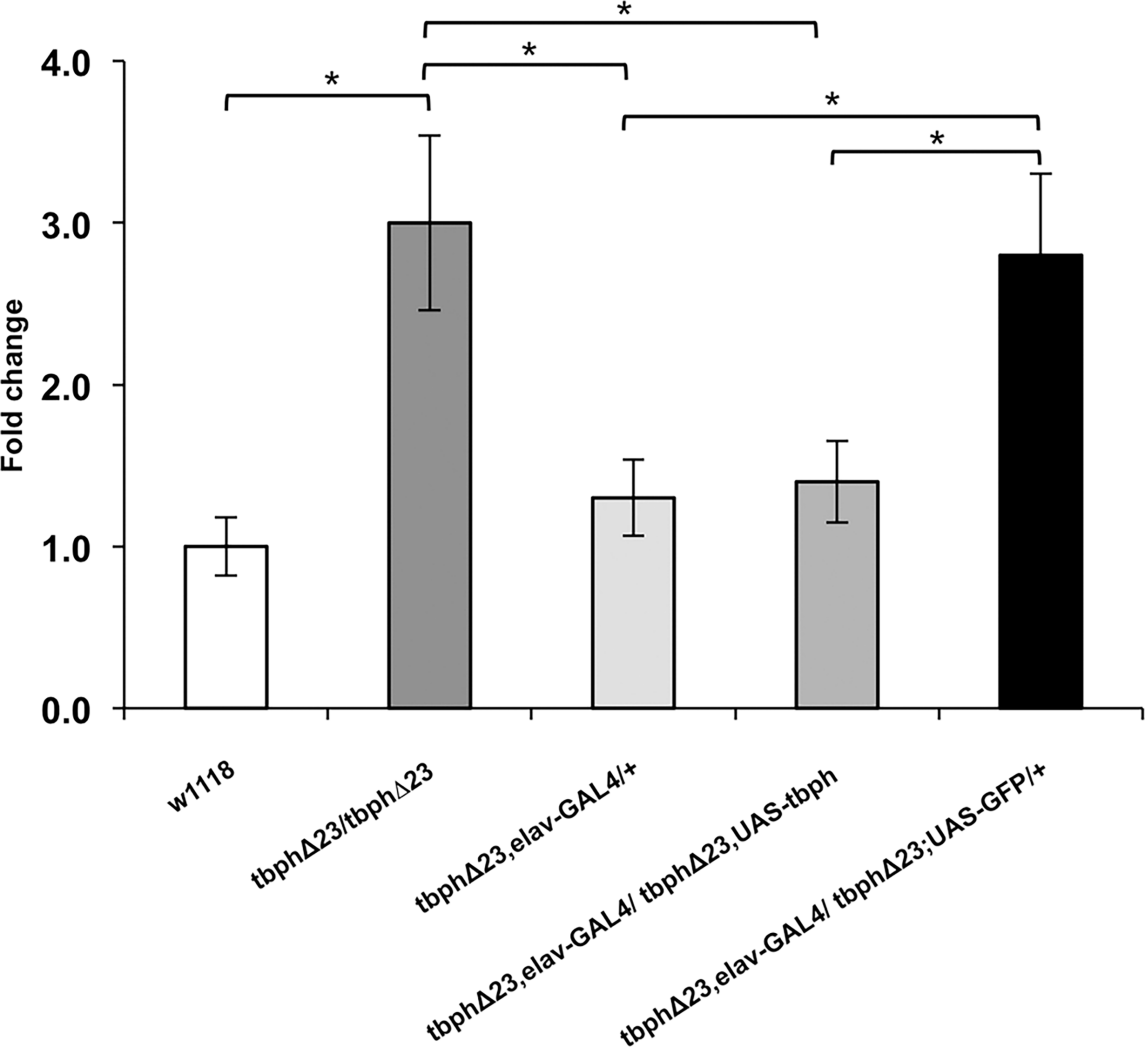
Correlation of RhoGAPp190 mRNA expression with tbph expression. Real Time PCR performed on fly heads: the mRNA levels are shown as relative expression vs w1118 genotype (= 1). In vivo tbph UAS-GAL4-driven overexpression in neurons (tbphΔ23, elav-GAL4/tbphΔ23,UAS-tbph) rescues RhoGAPp190 mRNA to the levels observed in control flies (tbphΔ23,elav-GAL4/+). GFP expression in tbph-null flies (tbphΔ23,elav-GAL4/tbphΔ23;UAS-GFP/+) was used as a control experiment. The expression in w1118 and tbph-null flies (tbphΔ23/tbphΔ23) is also shown. N = 3, *p<0.05 (t-test), error bars indicate SEM.

**Fig 3:**
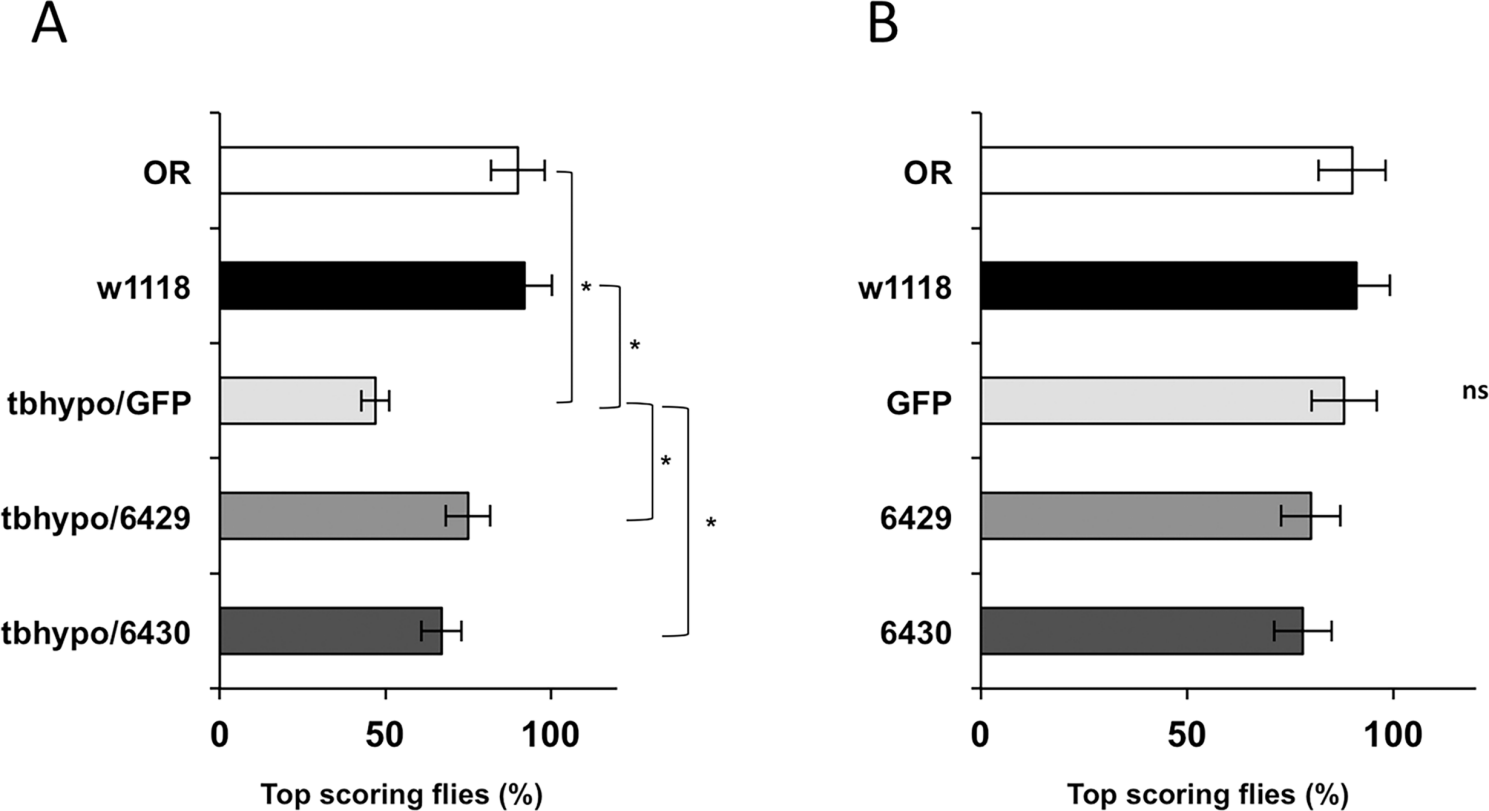
Genetic interaction between tbph and RhoGAPp190. A) The climbing ability of tbph-hypomorphic allele flies is partially rescued in flies silenced for RhoGAPp190 (tbhypo/6429: tbphΔ23,elav-GAL4/UAS-RhoGAPp190-RNAi;UAS-Dcr2,UAS-tbph-RNAi/+) and (tbhypo/6430: tbphΔ23,elav-GAL4/+;UAS-Dcr2,UAS-tbph-RNAi/UAS-RhoGAPp190-RNAi) flies with respect to the animals expressing an unrelated protein (tbhypo/GFP: tbphΔ23,elav-GAL4/+;UAS-Dcr2,UAS-tbph-RNAi/UAS-GFP). The assay was performed on 4 day-old adult animals. B) Effect of single RNAi overexpression in a wild type background (the behavior of w1118 and OregonR flies was overlapping). The observed variations were not statistically significant (ns). w1118 = white 1118. OR = OregonR. (GFP = elav-GAL4/+;UAS-GFP/UAS-Dcr-2). (6429 = elav-GAL4/RhoGAPp190 RNAi; UAS-Dcr-2/+). (6430 = elav-GAL4/+; RhoGAPp190 RNAi/Dcr-2). 100 flies for each genotype were tested. *p<0.05 (t-test), error bars indicate SEM.

To further verify whether tbph is involved in the regulation of RhoGAPp190 mRNA levels, we tested the effects of tbph overexpression in neurons of tbph-null flies on RhoGAPp190 transcripts level. To this end, the UAS-GAL4 system was used to drive ectopic expression of a gene of interest under the control of the GAL4 transcription factor [17]. In vivo elav-GAL4-driven tbph neuronal overexpression (tbphΔ23, elav-GAL4/ tbphΔ23,UAS-tbph) restored the RhoGAPp190 mRNA levels to that of the control flies (tbphΔ23, elav-GAL4/+) (Fig 2). As a further control, the unrelated protein GFP was expressed in the neurons of tbph-null flies (tbphΔ23, elav-GAL4/ tbphΔ23; UAS-GFP/+). Our results showed that GFP expression did not modify the RhoGAPp190 mRNA levels. These findings lend support to the hypothesis that RhoGAPp190 expression can be regulated by tbph. Subsequently, to test whether RhoGAPp190 mRNA upregulation can contribute to the locomotor dysfunction observed in the tbph-null flies, we assayed the genetic interaction between RhoGAPp190 and tbph by examining the consequences of the combined RhoGAPp190 / tbph silencing on climbing ability. Since flies bearing homozygous tbph-null mutations show limited larval lethality [15–18], tbph hypomorphic flies (carrying a milder phenotype) [19–22] were used to test the genetic interaction between this nuclear factor and RhoGAPp190. Two different RhoGAPp190 RNAi fly lines (namely, 6429 and 6430), generated by expressing RhoGAPp190 and tbph RNAi in tbph hypomorphic flies, were used for this experiment. In keeping with our thesis, the climbing ability of flies silenced for RhoGAPp190 expression (tbhypo/6429 and tbhypo/6430 lines) was significantly higher than that observed in the animals carrying only tbph hypomorphic allele alone (tbhypo/GFP) (Fig 3A). On the other hand, the climbing ability of flies was not significantly affected in the control flies carrying just RhoGAPp190 silencing (Fig 3B). These results suggest that a decrease of RhoGAPp190 expression is able to improve locomotion in tbph-depleted flies and support the presence of a genetic interaction between tbph and RhoGAPp190.

### Tbph protein binds the RhoGAPp190 mRNA

**Fig 4:**
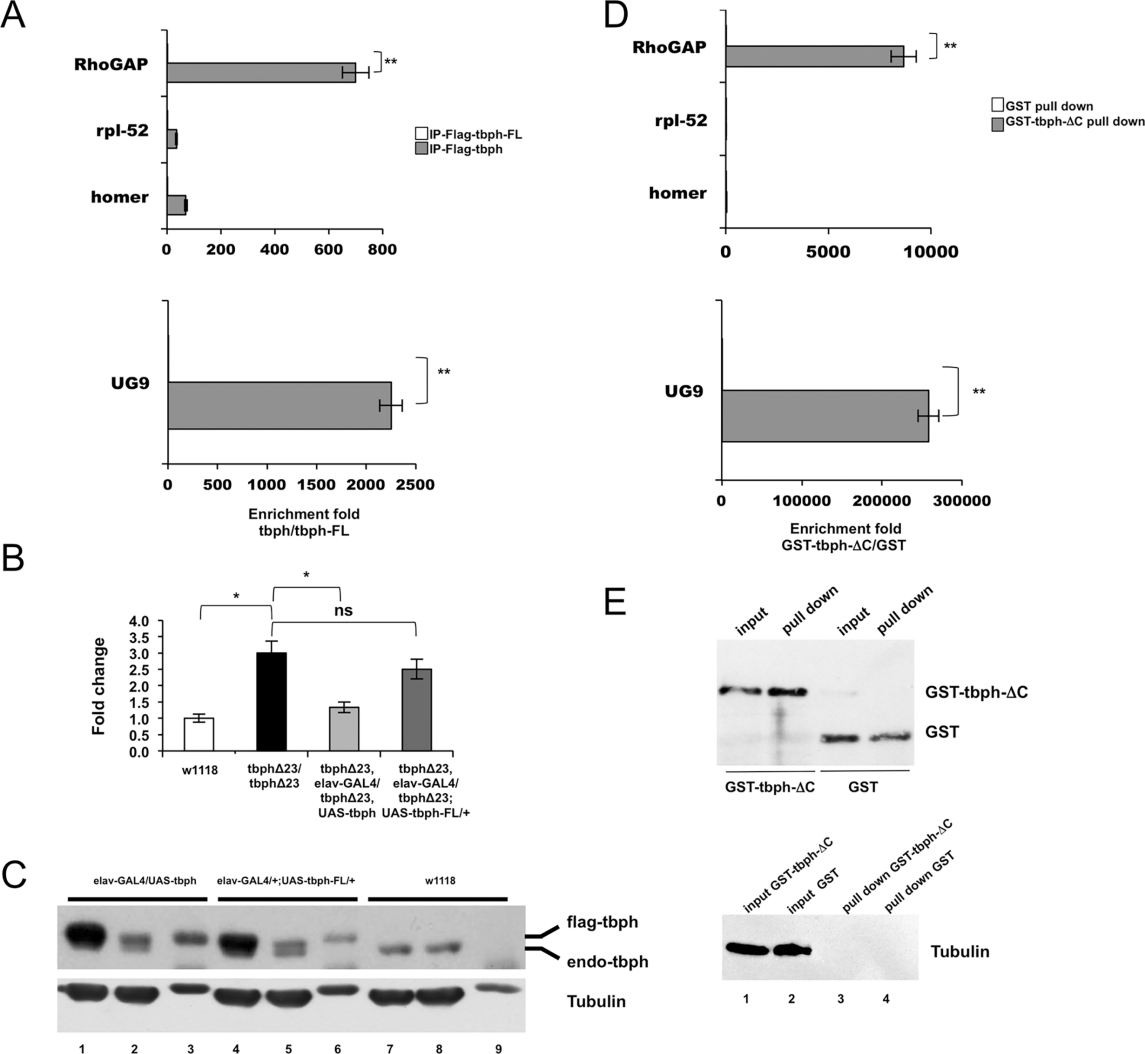
Probing tbph/RhoGAPp190 mRNA interaction. A) Co-immunoprecipitation. The RhoGAPp190 mRNA was immunoprecipitated with Flag-tagged tbph or with Flag-tagged tbph-FL from transgenic fly head extracts. The UG9 RNA was used as positive control. Homer and rpl-52 transcripts were used negative controls (not statistically significant). The enrichment-fold, quantified by Real Time PCR, is referred to the mutant tbph-FL (ratio tbph/tbph-FL). N = 3, **p<0.01 (t-test), error bars indicate SEM. B) Real Time PCR performed on fly heads to test the effects of tbph-FL overexpression on RhoGAPp190 mRNA expression: in vivo tbph overexpression in neurons (tbphΔ23,elav-GAL4/ tbphΔ23,UAS-tbph), by using the UAS-GAL4 system rescues the RhoGAPp190 mRNA levels to those observed in w1118 controls. The tbph-FL overexpression in tbph-null flies (tbphΔ23,elav-GAL4/ tbphΔ23;UAS-tbph-FL/+) did not significantly affect RhoGAPp190 expression. N = 3, **p<0.01 (t-test), error bars indicate SEM. C) Co-immunoprecipitation efficiency tested by Western blot. Head extracts from elav-GAL4/UAS-tbph, elav-GAL4/+;UAS-tbph-FL/+ and w1118 flies were probed with an anti-tbph antibody. Lanes 1, 4 and 7 = input; Lanes 2, 5 and 8 = supernatant not bound after incubation with beads/antibody; Lanes 3, 6 and 9 = co-IP samples. With the anti-tbph serum, both endogenous (endo-tbph) and flag-tagged tbph (flag-tbph) are visualized. D) GST pull-down. The RhoGAPp190 mRNA was precipitated with recombinant GST-tagged tbph or GST proteins. The UG9 RNA was used as positive control. Homer and rpl-52 transcripts were used as negative controls (not statistically significant). The enrichment-fold, quantified by Real Time PCR, is referred to the GST protein (ratio GST-tbph/GST). N = 3, **p<0.01 (t-test), error bars indicate SEM. E) GST pull down efficiency tested by Western blot. Head extracts from w1118 flies were probed with an anti-GST antibody before (input) and after pull down (pull down) with GST proteins. Western blot anti-Tubulin (Tubulin) was used as a loading control for input cell extracts.

To understand whether the functional link between RhoGAPp190 and tbph was direct, we tested the protein-mRNA physical interaction. Two different approaches (RNA-co-immunoprecipitation and RNA-GST-pull down) were used to test this hypothesis. The co-immunoprecipitation (co-IP) experiments were carried out with head extracts deriving from flies expressing a Flag-tagged form of wild -type tbph selectively in neurons, or from flies overexpressing tbph-FL, a variant of tbph mutated within the RNA binding domain [12] which does not significantly affect the expression of the target gene (Fig 4B). The levels of expressed or recombinant proteins used for these experiments were tested by immunoblot (Fig 4C and 4E). To support the specificity of the tbph-RhoGAPp190 mRNA interaction, accordingly to previous characterizations [12, 18], UG9 in vitro transcribed RNA as well as rpl-52 and homer transcripts were chosen as positive and negative controls, respectively. When the anti-Flag mAb was used in the IP step, a 700-fold enrichment of RhoGAPp190 mRNA was found in extracts from flies overexpressing tbph vs. extracts from flies overexpressing tbph-FL (Fig 4A, tbph/tbph-FL ratio). This enrichment was considered specific because of its significantly higher magnitude (>10-20-fold) than that observed for the unrelated genes rpl-52 and homer (Fig 4A). At the same time, as expected, the enrichment observed by qPCR for UG9 RNA was high (>2000-fold). Afterwards, the RNA-protein interaction was further probed by GST-pull down carried out by using purified recombinant GST-tbph-ΔC or GST proteins. The tbph-ΔC protein is a variant devoid of the C-terminal domain that retains RRM motifs and the ability to bind RNA at wild-type-comparable levels [8, 9, 22]. In this test, the qPCR showed that the RhoGAp190 mRNA was 8670-fold enriched (Fig 4D, tbph-ΔC/GST ratio). These results demonstrate that tbph binds the RhoGAPp190 mRNA and suggest that the lack of this interaction might be responsible for the increase of the RhoGAPp190 transcript levels observed in the tbph-null flies (and rescued by tbph overexpression).

### TDP-43 is implicated in the regulation of RhoGAPp190 expression

**Fig 5:**
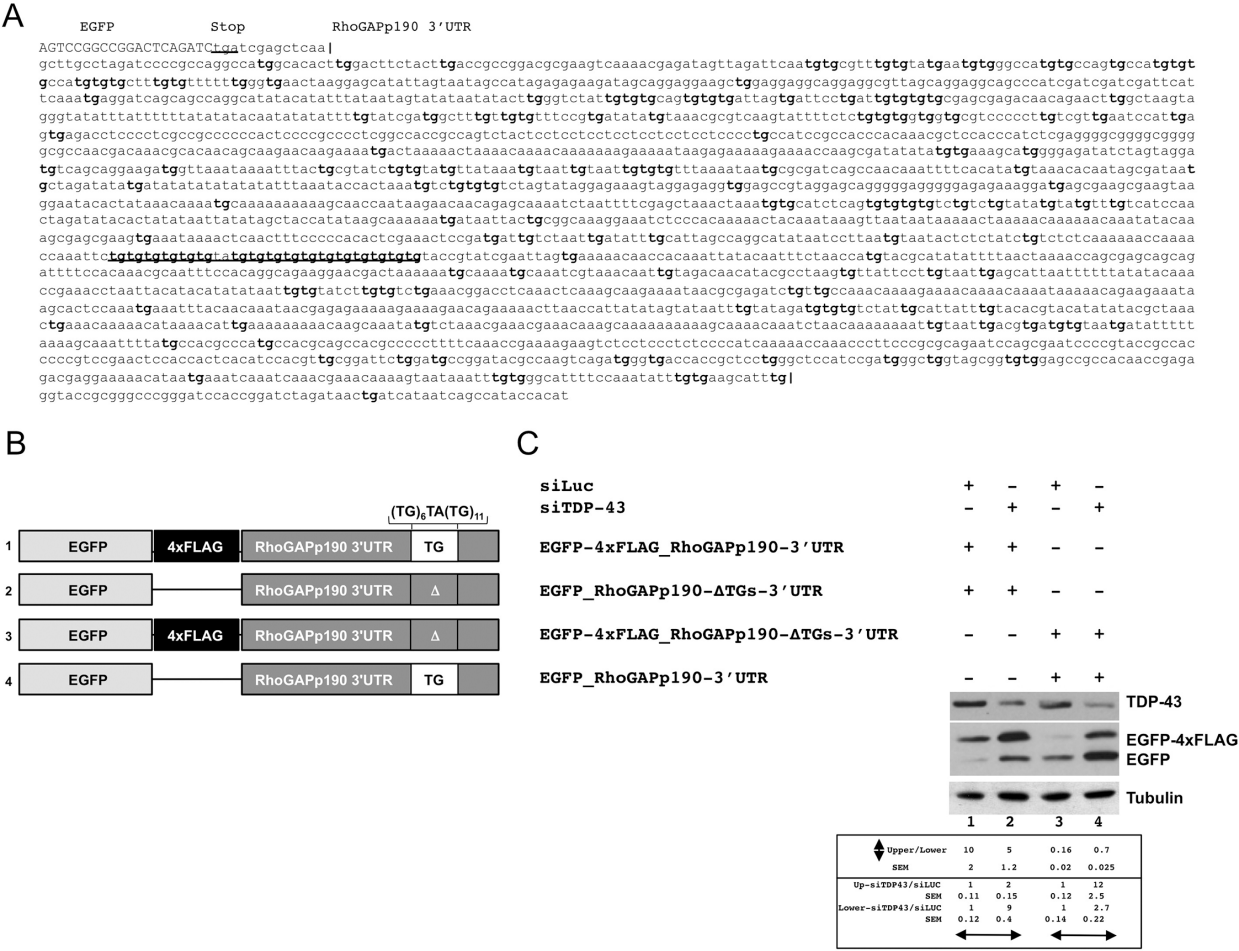
TDP-43 is implicated in the regulation of RhoGAPp190 expression. A) Sequence of the 3’UTR of RhoGAPp190 cloned in the pEGFP-C2 vector. All TG dinucleotide repeats within the 3'UTR are boldface. The TG-repeat rich sequence located 1470nts downstream of the stop codon is underlined. B) Schematic representation of the EGFP- RhoGAPp190-3’UTR constructs. All relevant elements within the construct are shown. C) Effect of TDP-43 depletion on EGFP-RhoGAPp190-3’UTR constructs. Equal amounts of the indicated EGFP-RhoGAPp190 constructs were co-transfected in Hek293 cells. The effects of TDP-43 silencing (siTDP-43) on EGFP expression were monitored by anti-GFP immunoblotting. Anti-luciferase siRNA (siLUC) was used as the control. Western blot anti-tubulin (Tubulin) served as the protein loading control. One representative blot is shown of three independent experiments. Quantification of protein levels are shown as relative ratio of upper vs lower band in each condition or of siTDP vs siLUC treatment. Values are expressed in mean (of three independent experiments) ± SEM. P<0.05.

RhoGAPp190 mRNA was initially identified as a possible target of tbph/TDP-43 binding because of the UG-repeats stretch (TG)6TA(TG)11 located 1470nt downstream of the stop codon (Fig 5A, underlined sequence). Given the known functional overlap between tbph and TDP-43, we tested the possible role of this region in mRNA stability in the human Hek293 cell line. To this end, the wild-type 2480bp RhoGAPp190 3’UTR was amplified by PCR and cloned downstream of the EGFP ORF in the pEGFP-C2 vector (EGFP- 4xFLAG_RhoGAPp190-3’UTR, Fig 5B, construct #1). We also generated a mutant version of this construct where the putative TDP-43 binding sequence ((TG)6TA(TG)11), located within the RhoGAPp190 3’UTR, was deleted (Fig 5B, construct #2, EGFP-RhoGAPp190-ΔTGs-3’UTR). In both cases, a translation stop codon was introduced upstream of the RhoGAPp190 3’UTR cloning site. The 4xFLAG tag was introduced downstream of the EGFP coding sequence in construct #1 (Fig 5B) to discriminate the EGFP products encoded by these two plasmids.

To investigate the role of TDP-43 in regulation of protein expression possibly dependent on the presence of the (TG)6TA(TG)11 sequence, equal amounts of these vectors were co-transfected in Hek293 cells after TDP-43 silencing by RNAi (siTDP-43). The RNAi against luciferase (siLUC) acted as the control condition. The anti-GFP immunoblotting showed that the EGFP expression derived from the ΔTG construct #2 (Fig 5C, lane 1, lower band) resulted significantly lower (10-fold) in comparison to that derived from the wild-type construct #1 (Fig 5C, lane 1, upper band). On the other hand, TDP-43 silencing caused a 2-fold increase of EGFP in the wild-type construct #1 (Fig 5C, compare the upper bands of lanes 1 and 2), and a 10-fold increase of EGFP in the mutant construct #2 (Fig 5C, compare the lower bands of lanes 1 and 2). It is interesting to note that the deletion of (TG)6TA(TG)11 sequence itself causes a reduction of EGFP levels (Fig 5C lane 1). In addition, considering the binding specificity of TDP-43 for UG-rich runs, these observations lend support to the hypothesis that this nuclear factor can be implicated in the regulation RhoGAPp190 expression by interacting with the 3'UTR of RhoGAPp190 mRNA. These conclusions were further validated by the results obtained by co-transfection of a second set of plasmids (EGFP-4xFLAG_RhoGAPp190-ΔTGs-3’UTR, Fig 5B, constructs #3 and #4), whereby the 4xFLAG tag was cloned within the RhoGAPp190-ΔTGs-3’UTR (EGFP-4xFLAG_RhoGAPp190-ΔTGs-3’UTR, Fig 5C, lanes 3 and 4). In fact, in the siLUC control transfection, the EGFP expression derived from the ΔTG construct #3 (Fig 5C, lane 3, upper band) resulted 10-fold lower, in comparison to that controlled by the wild-type construct #4 (Fig 5C, lane 3, lower band). Even with this second set of plasmids, TDP-43 silencing increased 10-fold the EGFP levels derived from the construct #3 (Fig 5C, compare the upper bands of lanes 3 and 4) and 2.5-fold the EGFP levels derived from the construct #4 (Fig 5C, compare the lower bands of lanes 3 and 4). On one hand, these results confirmed that the (TG)6TA(TG)11 sequence plays a critical role for EGFP expression. On the other hand, the persistence of response of the constructs #2 and #3 to TDP-43 silencing strongly suggests that the regulation of gene expression exerted by this nuclear factor relies on the presence of other TG-rich sequences located within the RhoGAPp190-3’UTR (Fig 5A). Although the detailed mechanisms of this phenomenon are not fully elucidated, these data support the hypothesis that RhoGAPp190 expression gene is negatively regulated by TDP-43/tbph and suggest that this factor exert its action by interacting with multiple TG-rich sites located within the RhoGAPp190 3'UTR, rather than with a single site.

## Conclusions

In the present manuscript, we identify the *Drosophila melanogaster* RhoGAPp190 factor as an additional player in the complex scenario of tbph-related neurodegeneration. Using a fly model lacking tbph expression, we provide evidence of the genetic interaction between tbph and RhoGAPp190. In addition, RhoGAPp190 seems to be linked to the TDP-43 protein through a regulatory function exerted by the interaction of this nuclear factor with the RhoGAPp190 transcripts. Interestingly, multiple UG-rich sequences, known as preferred binding sequence of tbph/TDP-43, are present within the RhoGAPp190 3’UTR. Apparently, the regulatory effect of TDP-43 on the fly RhoGAPp190 transcript is possibly mediated by these UG-rich sequences, as suggested by combining the results of in silico, in vivo, in vitro and ex vivo analyses. RhoGAPp190 is a Rho GTPase activating protein, initially identified as a binding partner of p120 RasGAP in Src-transformed cells [23, 24] and subsequently found to preferentially regulate the GTPase RhoA [25]. RhoGAPp190A is highly expressed in the developing and adult mammalian central nervous system, where it is involved in crucial developmental processes including neural tube closure, axon outgrowth, guidance and fasciculation [26]. In *Drosophila melanogaster*, RhoGAPp190 has also been shown to be involved in processes crucial for the proper functioning of nervous system, i.e. the regulation of axon branch stability [27], the control of motor axon defasciculation and target recognition during neuromuscular development [28].

This is particularly meaningful because of the established RhoGAPp190 involvement in processes that are crucial for proper synaptic contacts and nerve signalling [27] and the recent association of the human ortholog ARHGAP35 with progressive non-fluent aphasia, a subtype of frontotemporal dementia [29]. In addition, different lines of evidence support the connection of the human TDP-43 with the Rho family GTPases regulatory pathway of spinogenesis [30] and suggest that this nuclear factor might play a role in the maintenance of neuronal cell morphology and survival through protein geranylgeranylation of Rho-GTPases [31]. In conclusion, our results pinpoint the importance of RhoGAPp190 as an additional mediator of the neurodegenerative phenotype events associated with tbph loss-of-function and support the evolutionary conservation of the possible dysregulation of the TDP-43-Rho family GTPases pathway in the pathogenesis of TDP-43 proteinopathies.

## Materials and methods

### Transgenic flies, genetic rescue experiments and RNAi

The fly lines used are as follows:

(w1118), (OregonR), (w;tbphΔ23/CyOGFP) [15], (w;elav-GAL4/CyOGFP), (w;UAS-tbph) [15], (w;;UAS-GFP), (w;;UAS-Dcr-2), (w;;UAS-tbph-FL) [32], (w;;UAS-tbph RNAi (ID38377-VDRC)), (yw;UAS-RhoGAPp190 RNAi (#6429)), (yw;;UAS-RhoGAPp190 RNAi (#6430)). The other lines were obtained from the Bloomington *Drosophila* Stock Center. The RNAi efficiency in the used lines was confirmed by qPCR. The tbph RNAi efficiency in the used lines was previously tested [15].

### RNA extraction and quantitative real-time PCR (qPCR)

Adult fly males were collected within 24 hours after eclosion, frozen in liquid nitrogen and stored at -80°C. For RNA extraction heads and bodies were separated. Larval brains were dissected from third instar larvae. 10 *Drosophila* heads, bodies or larval brains/genotype were homogenized in 1 ml Trifast reagent (Euroclone, Milan, Italy) and RNA was extracted according to manufacturer’s instruction. The resulting RNA samples were digested with 1U of DNase RNase-free for 20 min at RT. For cDNA synthesis 500ng of RNA/sample were retrotranscribed with M-MLV reverse transcriptase (Invitrogen, Gaithersburg, MD, USA) and hexameric random primers or poly-(T)20 primer (Sigma-Aldrich, St. Louis, MO). Specific primer pairs were designed to analyze gene expression levels by qPCR, using sybr green technology (Bio-Rad Laboratories, Redmond, WA, USA). Expression of the house-keeping gene RpL32 was utilized to normalize gene expression of targets. qPCR amplifications were performed using a CFX96 real-time PCR detection system (Bio-Rad Laboratories, Redmond, WA, USA). The relative expression levels were calculated using the 2^-ΔΔCT method [33]. Statistical significance was calculated using unpaired t-test analysis. Values are presented as mean and error bars indicate standard errors of the mean (SEM). The results are representative of three independent experiments.

Primer pair sequences were the following: For RhoGAPp190, 5’-CGCCTGCCTCCGCTGTTC-3’; Rev RhoGAPp190, 5’-GTTGGAATATGTATTTGAGTATGG-3’; For RpL32, 5’-GCCCAGCATACAGGCCCAAG-3’; Rev RpL32, 5’-AAGCGGCGACGCACTCTGTT-3’.

The primer sequences for the other tested genes are available on request.

### Climbing assay

Newly eclosed flies were transferred to fresh feeding tubes (20 flies/genotype/tube). Four days later they were transferred, without anesthesia, to a 50 ml glass cylinder and their climbing ability was calculated by the percentage of flies able to reach the top of the cylinder in 15s. The test was repeated three times for each fly group and the final percentage of top-scoring flies was calculated from the resulting average values. A minimum of 60 flies/genotype were tested.

### Protein-RNA co-immunoprecipitation and GST pull down

After rinsing protein G magnetic beads (Invitrogen, Gaithersburg, MD, USA) with PBS+0.02% Tween, anti-FLAG M2 monoclonal antibody (Sigma-Aldrich, St. Louis, MO) was added to the beads. Fly heads (elav-GAL4/UAS-tbph—elav-GAL4/+;UAS-tbph-FL/+ and w1118) extracts were prepared by homogenization in Lysis buffer (20 mM Hepes, 150 mM NaCl, 0.5 mM EDTA, 10% Glycerol, 0.1% Triton X-100, and 1 mM DTT) followed by centrifugation for 5 min at 0.4 rcf. The (UG)9 RNA [32, 34] was added to the head extracts in order to control the efficiency of immunoprecipitation (specific vs non-specific). Pretreated beads and head extracts were mixed and incubated for 60 min at 4°C, followed by washing five times with lysis buffer. Bound RNA transcripts were pooled with DynaMag-Spin (Invitrogen, Gaithersburg, MD, USA) and purified with Trifast (Euroclone, Milan, Italy). Synthesis of cDNA was carried out with M-MLV (Invitrogen, Gaithersburg, MD, USA) and Real Time PCR was performed with gene specific primers. The used primers are the following:

homer: 5′-GGTATAAACTGCTGCGGAAG-3′, and 5′-GACACTGATGATGCGGTAC-3′.

rpl-52: 5′-GAAAATAACAAAGATCTGCTTGGCC-3′ and 5′-AAGTGGCCCTTGGGCTTCAG-3′.

The pBSKS 929_950as oligo 5′-AGCGGGCAGTGAGCGCAACGCA-3′ was used for specific reverse transcription of (UG)9 RNA. Amplification of (UG)9 transcripts was carried out with the following oligos: pBSKS 667_687s 5′-TGGCGGCCGCTCTAGAACTA-3′ and pBSKS 903_924s 5′-ATGTGAGTTAGCTCACTCATTA-3′.

The enrichment fold was calculated by normalizing all data vs the respective inputs then followed by the subtraction of controls Δub (tbph-FL or GST) from GST) n experimental sample overexpressing tbph-wt or w1118 flies. The results were derived from three independent immunoprecipitation experiments and error bars represent standard errors. The statistical significance of differences observed between control- and specific- immunoprecipitation samples were determined by t-test.

For GST-pull down, 30μl/sample of GSH beads (GE Healthcare Bio Sciences, AB, Sweden) were used. Beads were pre-cleared by washing twice with 1 ml of HEGN buffer (20 mM Hepes pH 7.7, 150 mM NaCl, 0.5 mM EDTA, 10% Glycerol, 0.1% Triton X-100, 1mM DTT, Protease inhibitor cocktail 1x). 5 μg of each GST-fusion protein was added to the washed beads in a final volume of 1000 μl of HEGN buffer containing 0.5 mg/ml BSA. After 2 hrs of incubation at 4°C, the mix was washed 5 times with HEGN buffer (spin down beads at 3k, 5 min). Then, the GST-resin pellet was resuspended in Trifast reagent (for RNA extraction purposes) or in SDS sample buffer (for Western blot purposes). The data presented are representative results of three independent experiments. The statistical significance of differences observed between control- and specific- precipitation samples were determined by t-testing.

For protein analysis, extracts and supernatants were diluted in Laemmli buffer (0.1 M Tris-HCl pH 6.8, 30% (v/v) glycerol, 8% (w/v) SDS, 9.8% (v/v) β mercaptoethanol and 0.1% (w/v) bromophenol blue), boiled at 95°C for 5 min and loaded in 15% SDS-PAGE. Protein samples were transferred to Whatman Protran nitrocellulose blotting membranes (Fisher Scientific, Hanover Park, IL, USA) and probed with primary antibodies: rabbit anti-tbph (1:1500, home made), mouse anti-tubulin CP06 (1:4000, Calbiochem, San Diego, CA, USA). The membranes were incubated with the secondary antibodies: HRP-labeled anti-mouse or anti-rabbit (1:1000, Thermo Fisher Scientific, Waltham, MA, USA). An anti-GST antibody, HRP Conjugate (1:1000, GE Healthcare Bio Sciences, AB, Sweden) was used to detect GST fusion proteins. Protein detection was assessed with ECL Western Blotting Substrate (Thermo Fisher Scientific, Waltham, MA, USA).

### Expression plasmids, HEK293 transfection and immunoblotting

Expression plasmids were obtained by cloning the sequences of interest in the pEGFP-C2 vector (Clontech, Palo Alto, CA, USA). RhoGAPp190 3’UTR sequence was amplified by PCR from *Drosophila melanogaster* genomic DNA using the following primer couple:

RhoGAPp190_3UTR_SacI/HindIII_For, 5’-ACGGAGCTCAAGCTTGCCTAGATCCCCGCCAGGCCATGGCA-3’; RhoGAPp190_3UTR_EcoRI/KpnI_Rev, 5’-ACGGAATTCTGCAGTCGACGGTACCCAAATGCTTCACAAATATTTGGAAAAT-3’. The restriction enzymes used in the 3’UTR cloning step were SacI and KpnI. Upstream of the SacI cloning site a stop codon for EGFP protein expression was inserted by quick-change site-directed mutagenesis (Stratagene, La Jolla, CA, USA), according to manufacturer’s instructions, by using the following primer couple:

EGFPstopRho_For, 5’-GGCCGGACTCAGATCTGATCGAGCTCAAGCTTGC-3’;

EGFPstopRho_Rev, 5’-GCAAGCTTGAGCTCGATCAGATCTGAGTCCGGCC-3’.

When required, a 4xFLAG-tag coding sequence followed by its own stop codon was inserted downstream of the EGFP ORF, by using the Sac I cloning site. Cloning directionality was checked by sequencing. Quick-change site-directed mutagenesis [35] was also used to generate the TGs-deleted version of plasmids by deleting the (TG)6TA(TG)11 from the RhoGAPp190 3’UTR sequence. To this aim the following primer couple was used:

ΔTGs_RhoGAPp190_3UTR_For,

5’-TCTGTCTCTCAAAAAACCAAAACCAAATTCTACCGTATCGAATTAGTGAAAAACAACCAC-3’;

ΔTGs_RhoGAPp190_3UTR_Rev,

5’-GTGGTTGTTTTTCACTAATTCGATACGGTAGAATTTGGTTTTGGTTTTTTGAGAGACAGA-3’.

The 4 resulting expression plasmids were the following: EGFP-RhoGAPp190-3’UTR, EGFP-RhoGAPp190-ΔTGs-3’UTR, EGFP-4xFLAG_RhoGAPp190-ΔTGs-3’UTR, EGFP-4xFLAG_RhoGAPp190-3’UTR (their schematic representation is shown in Fig 5B).

Hek293 cells were cultured in Dulbecco’s modified Eagle’s medium (DMEM, Invitrogen, Gaithersburg, MD, USA) medium supplemented with 10% (v/v) heat inactivated fetal bovine serum (FBS) and 1X antibiotic-antimycotic (Sigma-Aldrich, St. Louis, MO). For transfection assays, one day before transfection 400,000 cells were seeded in each well of a 6-well plate. Cells were transfected (2 μg plasmid /well) with Effectene reagent (Qiagen, Cologne, Germany), according to the manufacturer’s instructions, and were then incubated at 37°C in a CO_2_ incubator for 48 hrs, prior to transgene expression testing. For protein analysis, cells were harvested and lysated with Lysis buffer (10% (v/v) glycerol, 50 mM Tris HCl pH = 7.5, 150 mM NaCl, 1% (v/v) TritonX100 and protease inhibitors (Roche Diagnostics, Mannheim, Germany). Cells were sonicated for 10 min (sonication cycles: 30 sec on/30 sec off) at medium intensity (Bioruptor, Diagenode Inc, Denville, NJ, USA). Then, the solution was centrifuged at 1000 x g for 10 min at 4°C. 12μl of the resulting supernatant protein samples were diluted in Laemmli buffer (0.1 M Tris-HCl pH 6.8, 30% (v/v) glycerol, 8% (w/v) SDS, 9.8% (v/v) β-mercaptoethanol and 0.1% (w/v) bromophenol blue), boiled at 95°C for 5 min and loaded in 15% SDS-PAGE. Protein samples were transferred to Whatman Protran Nitrocellulose blotting membranes (Fisher Scientific, Hanover Park, IL, USA) and probed with primary antibodies: mouse anti-GFP (1:1000, Roche Diagnostics, Mannheim, Germany), mouse anti-tubulin (1:4000, Calbiochem, San Diego, CA, USA). The membranes were incubated with the secondary antibody: HRP-labeled anti-mouse (1:1000, Thermo Fisher Scientific, Waltham, MA, USA). Protein detection was assessed with ECL Western Blotting Substrate (Thermo Fisher Scientific, Waltham, MA, USA). Protein bands were quantified using NIH ImageJ software [36]. The average ratio values of all blots (three independent experiments) were expressed as a relative increase (or decrease) in protein expression between "experimental" and "control" groups.

## Supporting information

S1 Table ENSEMBL fly transcripts and genes with (TG)_≥6_ within 3'UTR. List of all the Drosophila melanogaster transcripts and genes containing at least 6 (TG) repeats in a row within their 3’UTR region, identified in the ENSEMBL Genes 86 database.
(XLSX)
